# Dual role of IL-17A in COPD: amplifier of inflammatory cascades and mediator of airway remodeling and alveolar destruction

**DOI:** 10.3389/fimmu.2026.1828172

**Published:** 2026-05-29

**Authors:** Lanying Zhang, Yanhui Gu, Renli Deng, Yao Ouyang

**Affiliations:** 1 Department of Respiratory and Critical Care Medicine, Affiliated Hospital of Zunyi Medical University, Zunyi, China; 2 Department of Nursing, Affiliated Hospital of Zunyi Medical University, Zunyi, China

**Keywords:** airway remodeling, chronic obstructive pulmonary disease, corticosteroid resistance, IL-17A, neutrophilic inflammation, precision medicine

## Abstract

Corticosteroid resistance remains a central challenge in managing chronic obstructive pulmonary disease (COPD). This refractory phenotype is primarily driven by persistent, neutrophil-dominated airway inflammation. Interleukin-17A (IL-17A) bridges innate and adaptive immunity and helps sustain this refractory inflammation, although it operates within a redundant cytokine network and its pathogenic contribution is clearest in a defined molecular subset of patients. Following an overview of upstream drivers including lung-gut microbiome dysbiosis and Th17/Treg immune imbalance, the downstream effector network of IL-17A is analyzed. In sustaining inflammation, IL-17A stabilizes pro-inflammatory transcripts via ACT1-mediated post-transcriptional regulation and produces a self-amplifying positive feedback loop with neutrophil extracellular traps (NETs). In tissue remodeling, IL-17A induces alveolar epithelial ferroptosis via the ACT1-TRAF6-p38 MAPK cascade to drive emphysema. It also mediates irreversible structural alterations in the airway and alveolar parenchyma by inhibiting fibroblast autophagy through the PI3K/AKT/mTOR pathway and inducing epithelial mucus hypersecretion. Given the lack of significant clinical benefit from early non-selective IL-17A blockade in unselected populations, precision intervention strategies guided by clinical endotypes are evaluated. Optimizing next-generation targeted therapies in COPD necessitates biomarker-driven patient stratification, coupled with upstream signal interception and the restoration of systemic immune homeostasis. Together, these strategies support a shift from symptomatic management toward endotype-specific disease modification.

## 1 Introduction

The clinical management of chronic obstructive pulmonary disease (COPD) remains a significant challenge. According to the latest Global Burden of Disease (GBD) predictive models published in *The Lancet Respiratory Medicine*, the epidemiological trajectory of COPD is shifting. By 2050, the global number of patients is projected to approach 600 million, with the disease burden increasing most rapidly among women and populations in low- and middle-income countries (1). For this large and heterogeneous population, standard anti-inflammatory therapies based on inhaled corticosteroids (ICS) show limited efficacy in preventing disease progression and reducing mortality (2, 3). Absolute measures of clinical benefit, including the number needed to treat (NNT), indicate that only patients with specific phenotypes derive substantial benefit from current intensified treatments (4).

A major challenge in clinical practice is that many severe patients fail to benefit from standard ICS therapy. The airway microenvironment in this refractory subpopulation is typically characterized by neutrophil infiltration and persistent corticosteroid resistance (5). Simple ICS dose escalation primarily increases the risk of secondary local infections due to non-specific immunosuppression rather than conferring clinical benefit (6, 7). This corticosteroid-resistant phenotype is closely associated with accelerated lung function decline, frequent exacerbations, and irreversible airway remodeling, highlighting the need to identify new therapeutic targets for early disease interception (8). The key clinical, structural, and immunological features of COPD relevant to this review are summarized in **Table 1** (9–12). The term “airway remodeling” in COPD encompasses a broad spectrum of structural changes. In this review, the term refers specifically to the inflammatory and fibrotic alterations of the airway wall that are potentially modifiable by therapeutic intervention, including subepithelial fibrosis, collagen deposition, goblet cell metaplasia, epithelial-mesenchymal transition (EMT), and airway smooth muscle (ASM) changes. This is distinct from the irreversible physical obliteration and disappearance of terminal bronchioles, which is a separate pathological endpoint (13, 14). COPD pathology extends well beyond bronchiolitis and emphysema. It also includes mucus plugging as an independent driver of disease progression (15), pulmonary vascular remodeling, and systemic comorbidities (1, 8). This structural heterogeneity is compounded by endotypic diversity: IL-17A-driven pathology represents only one of several disease trajectories (5), with other subgroups driven by type 2 eosinophilic inflammation, CD8^+^ T cell cytotoxicity, or protease-antiprotease imbalance within a redundant inflammatory network. This suggests that alternative molecular networks independent of the classical glucocorticoid receptor (GR) pathway drive this refractory inflammation (16–18).

**Table 1:** Overview of clinical, structural, and immunological features of COPD.

Domain	Key features	References
Clinical Presentation	Progressive dyspnea, chronic cough, sputum production; poorly reversible airflow limitation (post-BD FEV_1_/FVC <0.70); recurrent exacerbations; systemic comorbidities (cardiovascular disease, skeletal muscle dysfunction, osteoporosis)	(1, 8)
Airway Structural Components and Pathological Alterations	Components: pseudostratified epithelium (ciliated, goblet, basal, Club cells), basement membrane, subepithelial ECM (collagens I/III/V, proteoglycans, elastic fibers), ASM, submucosal glands, peribronchial vasculature COPD alterations: ASM hypertrophy/hyperplasia (~50% mass↑in GOLD 3–4, inversely correlates with FEV_1_); submucosal gland hypertrophy (↑Reid index); RBM thickening/fragmentation; subepithelial fibrosis with elastic fiber loss; peribronchial neovascularization	(92, 9–12)
Epithelial Alterations	Goblet cell metaplasia; ciliated cell loss with mucociliary dysfunction; squamous metaplasia; tight junction disruption (ZO-1, claudin-1); oxidative DNA damage and senescence; alveolar epithelial ferroptosis (GPX4↓)	(82, 43, 85)
EMT	Loss of epithelial polarity with ↓E-cadherin and ↑Vimentin/α-SMA, contributing to subepithelial fibrosis and airway wall thickening; Key drivers: TGF-β, CXCL12/CXCR4, IL-17A/ADAM9	(28, 89)
Innate Immune Mechanisms	Neutrophil-dominant inflammation with NETosis; M1 macrophage activation (MMP-9/12, ROS) with impaired efferocytosis; DC dysregulation (SOCS1↓, Th17 skewing); ILC3-derived IL-17A amplifying neutrophil recruitment	(54, 55, 16, 52, 126)
Adaptive Immune Mechanisms	CD8^+^ T cell cytotoxicity (perforin/granzyme); Th17/Treg imbalance (Th17 expansion, Treg exhaustion); Th1/IFN-γ bias; ectopic lymphoid follicle formation	(51, 75, 124)
Corticosteroid Resistance	Post-transcriptional mRNA stabilization (ACT1/Arid5a bypassing GR transrepression); HDAC2 impairment via oxidative stress and NETs; GRα↓/GRβ↑	(69, 3, 16, 87)

Translational evidence points to IL-17A as a key amplifying node in this process (19), although transcriptomic data suggest that an IL-17A-responsive epithelial gene signature is present in a minority of COPD patients (5). Linking innate and adaptive immunity (20, 21), aberrant IL-17A overexpression in the COPD lung positively correlates with disease severity and lung function decline (22). Single-cell and transcriptomic studies confirm that specific γδT cell subsets, including clonal γδT17 cells, are key local sources of IL-17A (23, 24). Distinct from classical type 2 inflammation, IL-17A forms an auto-amplification circuit centered on neutrophils. It induces effector cell chemotaxis by upregulating CXCL1 and CXCL8 and utilizes unique post-transcriptional mechanisms to stabilize pro-inflammatory mRNAs (25). IL-17A and neutrophil extracellular traps (NETs) form a highly coupled positive feedback loop that amplifies local inflammation (26). This persistent neutrophilic inflammation often spills over into the systemic circulation, increasing the risk of neuropsychiatric comorbidities including cognitive impairment (27). IL-17A directly mediates EMT and extracellular matrix (ECM) deposition via the CXCL12/CXCR4 axis (28) or the PI3K/AKT/mTOR pathway (29), ultimately solidifying irreversible airflow limitation (30).

The pathogenic network of IL-17A is governed by multiple environmental and microbiome factors (31, 32). In addition to air pollutant exposure including PM2.5 (33), recent studies on the gut-lung axis indicate that respiratory microbiome dysbiosis (specifically aberrant colonization of *Moraxella* and *Haemophilus influenzae*) acts as an initiating factor driving high local IL-17A levels and frequent exacerbations (34, 35). Cross-organ models confirm that gut microbial metabolites, including short-chain fatty acids (SCFAs), act as epigenetic regulators to remotely suppress excessive Th17 activation in the lungs (36–38). Local epigenetic modifications, including histone demethylation, directly promote pathogenic Th17 generation at the transcriptional level (39). This continuous release of IL-17A is compounded by aging-related immunometabolic disorders including nicotinamide adenine dinucleotide (NAD^+^) depletion (40), disrupting systemic Th17/Treg homeostasis (18, 41). This immunological instability compromises basal host defense against pathogens through epithelial barrier dysfunction (15, 42) and triggers severe tissue destruction via uncontrolled lipid peroxidation and ferroptosis pathways within a sterile microenvironment (43).

Although IL-17A-targeted biologics demonstrate efficacy in diseases like psoriasis, their early clinical trials in COPD including the CNTO6785 study encountered translational bottlenecks (44). This setback highlights the high heterogeneity of COPD, suggesting that non-selective pan-blockade strategies are inadequate and that only patients with specific phenotypic traits, including non-type 2 inflammation or high neutrophil burden, possess the potential for clinical benefit (5, 45). Future development pathways must transition toward biomarker-driven precision medicine stratification. This review examines the pivotal mechanisms of IL-17A within the inflammatory and remodeling networks of COPD and evaluates therapeutic strategies ranging from upstream microbiome interventions (31, 38) and systemic immunomodulation using traditional Chinese medicine formulas (46–48) to precise downstream signal blockade (49, 50). This analysis provides a theoretical foundation for the development of next-generation disease-modifying drugs for COPD.

## 2 The IL-17A-driven pathological network in COPD: From molecular mechanisms to targeted interventions

In COPD, chronic airway inflammation progressively leads to irreversible structural remodeling. IL-17A signaling serves as a key convergence point in this process, linking persistent neutrophilic infiltration to corticosteroid-resistant tissue damage. As noted in the Introduction, this pathogenic contribution is confined to a molecularly defined patient subpopulation; the majority of COPD patients progress through IL-17A-independent pathways including CD8^+^ T cell-mediated cytotoxicity and protease-antiprotease imbalance. To provide a basis for targeted therapies, this section reviews the pathological mechanisms of IL-17A, detailing its upstream immunological triggers and its downstream effects on airway fibrosis and emphysema.

### 2.1 Core signaling pathways and regulatory mechanisms of IL-17A

The sustained activation of IL-17A in COPD involves both its continuous upstream production and its specific downstream intracellular signaling. Identifying therapeutic targets requires a detailed understanding of these processes. The subsequent subsections examine how endogenous immune networks and epigenetic factors drive Th17 polarization and IL-17A expression, how microbiome dynamics and the gut-lung axis modulate this process, and how post-receptor signaling pathways bypass standard glucocorticoid suppression.

#### 2.1.1 Upstream cytokine networks and epigenetic drivers of Th17 polarization

Cytokine networks and epigenetic modifications synergistically drive aberrant IL-17A expression in the COPD airway. Th17 differentiation and expansion depend on combined signals from IL-6 and TGF-β, with IL-23 serving as an essential signal for maintaining the pathogenic Th17 phenotype (51). IL-1β induces type 3 innate lymphoid cells (ILC3s) to secrete IL-17A, further amplifying neutrophilic inflammation (52). Translational evidence using human lung tissue explants corroborates these immune-driven pathways, demonstrating that cigarette smoke (CS) exposure directly prompts local pulmonary structural cells to upregulate IL-17A and IL-17F expression via the PI3K and NF-κB signaling axes (53).

The phenotypic dysregulation of antigen-presenting cells, particularly dendritic cells (DCs), drives the Th17/Treg imbalance. CS exposure disrupts the equilibrium between mature and immature DCs in the airway, compromising DC tolerance by downregulating negative regulators such as SOCS1 and directly driving naive T cell polarization toward a Th17 phenotype (54, 55). During this polarization, histone deacetylase (HDAC)-mediated chromatin remodeling (17) and Notch-Hes1 signaling hyperactivation (56, 57) drive *IL17A* transcription. Blocking Notch signaling or its downstream PTEN/AKT circuit reverses this polarization process (58).

While these mechanisms drive Th17 expansion, the opposing arm of immune regulation is simultaneously weakened. Under persistent oxidative stress, Treg cells become phenotypically unstable, losing *Foxp3* expression and transdifferentiating into Th17-like cells (51). This transdifferentiation, compounded by the de-repression of Notch signaling resulting from downregulated long non-coding RNA *PVT1* expression (59), aggravates systemic Th17/Treg imbalance (60). IL-17A normally induces IL-24 to form a self-limiting negative feedback loop (61, 62). Under chronic stress, this defense mechanism becomes dysregulated, leading to sustained activation of the downstream PI3K/AKT/mTOR pathway and subsequent airway remodeling. Restoring the upstream immune-metabolic microenvironment or targeting specific antigen-presentation nodes thus offers disease-modifying potential beyond cytokine neutralization.

#### 2.1.2 Microbiome dynamics and the gut-lung axis in IL-17A regulation

Airway and gut microbiota dynamics serve as key exogenous drivers of this pathway abnormality. Clinical evidence indicates that aberrant airway colonization by specific bacteria, including *Moraxella* and *Lactobacillus*, relieves the endogenous post-transcriptional inhibition of IL-17A by downregulating non-coding RNAs such as *miR-122* and *miR-30a (*31). In gut-lung axis crosstalk, gut microbial metabolites such as SCFAs act as natural HDAC inhibitors. These metabolites maintain a hyperacetylated state in the *Foxp3* promoter region, antagonizing RORγt transcriptional activity and inhibiting IL-17A expression at the chromatin level (37, 38). Microbial dysbiosis impairs this remote immunological counterbalance (36).

#### 2.1.3 Downstream signal transduction and post-receptor escape mechanisms

IL-17A signals through high-affinity binding to the heterodimeric receptor IL-17RA/RC, recruiting the adaptor protein ACT1 via the intracellular SEFIR domain (17). This binding triggers an intracellular bifurcation in downstream signaling. In the classical transcriptional initiation branch, ACT1 recruits TRAF6 to activate the NF-κB and MAPK (p38, JNK, ERK) cascades, mediating the primary transcription of pro-inflammatory genes (21). Because the transcriptional response induced by IL-17A alone is relatively weak, its pathogenic efficacy stems from molecular synergy with TNF-α, IL-1β, or TLR3 ligands such as Poly I:C (63). This synergy operates through bidirectional crosstalk between IL-17A and NF-κB signaling. In the forward direction, TNF-α and IL-1β activate NF-κB through their respective receptor-proximal cascades (TNFR/TRADD and IL-1R/MyD88), providing convergent yet non-redundant inputs (52). These co-stimulatory signals initiate primary NF-κB-dependent transcription, and IL-17A then amplifies this output through a post-transcriptional mechanism: TNF-α-activated NF-κB transcribes the gene encoding the atypical cofactor IκBζ (NFKBIZ), while IL-17A stabilizes IκBζ mRNA by suppressing Regnase-1-mediated degradation. Accumulated IκBζ protein associates with DNA-bound NF-κB and C/EBPβ to induce ELF3, which activates secondary response genes including CSF3, CXCL5, and multiple MMP genes (64). This IκBζ-ELF3 cascade explains why IL-17A/TNF-α co-stimulation is far more potent than either cytokine alone, consistent with the enhanced IκB-α phosphorylation observed during IL-17A synergism in airway epithelial cells (63). A separate mechanism reinforces IL-6 production. The adaptor protein 14-3-3ζ sequesters TRAF5 and lifts its inhibition of IL-6 expression (65), adding a second layer to the same amplifying loop.

In the reverse direction, NF-κB also drives local IL-17A production, as pharmacological NF-κB inhibition attenuates cigarette smoke-induced IL-17A/F expression in human lung tissue explants (53). This produces a closed bidirectional circuit that is further sustained by IL-6-mediated STAT3 activation: NF-κB-dependent IL-6 activates STAT3, which prolongs NF-κB nuclear retention and sustains IL6 transcription (66). The continued operation of this loop in the COPD airway depends on IL-17A-mediated IκBζ mRNA stabilization (64), linking it to the post-transcriptional escape mechanism described below. Consequently, the synergistic interplay between these pathways upregulates key neutrophil chemokines including CXCL8, CXCL1, and CSF3, driving the continuous recruitment of effector cells and the formation of NETs (16, 62, 64). The local expression levels of these chemokines correlate with the frequency of COPD exacerbations (67).

**Figure 1:**
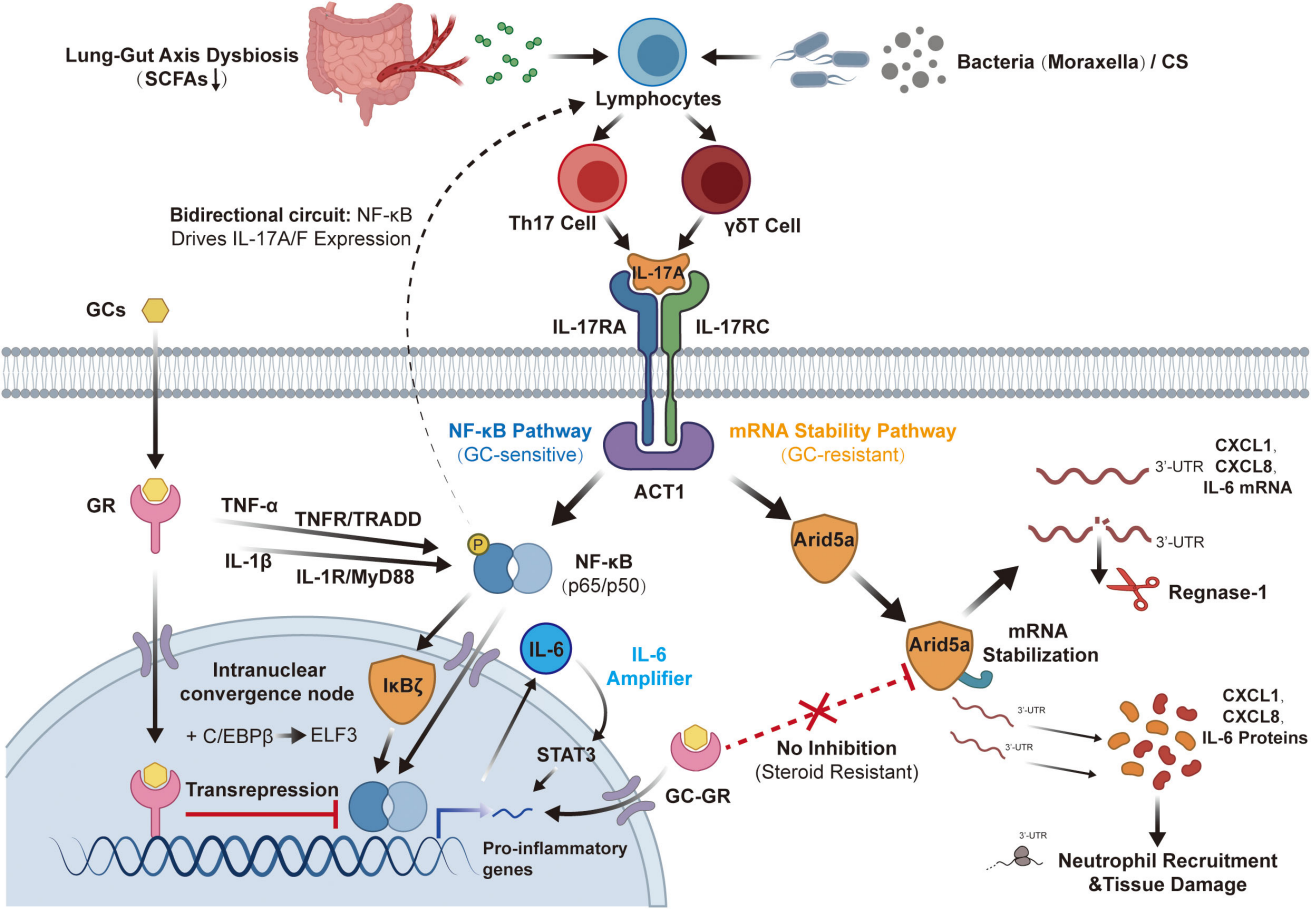
The molecular landscape of IL-17A-driven inflammation and mechanisms of corticosteroid resistance in COPD. Environmental triggers including bacteria and cigarette smoke (CS), alongside gut-lung axis dysbiosis, induce Treg inhibition and drive IL-17A production from Th17 and γδ T cells. Upon binding to the IL-17RA/RC complex, the adaptor protein ACT1 orchestrates an intracellular signaling split. (Left) The classical NF-κB transcriptional pathway operates through bidirectional crosstalk with IL-17A signaling. TNF-α (via TNFR/TRADD) and IL-1β (via IL-1R/MyD88) provide convergent NF-κB activation, initiating primary transcription. IL-17A amplifies this output: accumulated IκBζ associates with NF-κB and C/EBPβ to induce ELF3, activating secondary response genes. NF-κB-dependent IL-6 activates STAT3, which prolongs NF-κB nuclear retention, establishing an IL-6 amplifier loop. In the reverse direction, NF-κB drives local IL-17A/F expression, completing a bidirectional self-perpetuating circuit. This transcriptional branch remains sensitive to glucocorticoid receptor (GR)-mediated transrepression. (Right) Conversely, the corticosteroid-resistant pathway is driven by ACT1-mediated signaling, promoting the stabilization of pro-inflammatory mRNAs through the RNA-binding protein Arid5a. Arid5a competitively displaces the ribonuclease Regnase-1, preventing mRNA degradation. These two branches operate in parallel: the NF-κB/IκBζ transcriptional loop generates new pro-inflammatory transcripts, while ACT1/Arid5a-mediated mRNA stabilization preserves the existing cytoplasmic mRNA pool. Since GR transrepression acts primarily at nuclear transcriptional initiation, neither layer is effectively suppressed by corticosteroids, establishing the molecular basis for corticosteroid insensitivity in neutrophilic COPD. ACT1, Activating transcription factor 1; Arid5a, AT-rich interactive domain-containing protein 5A; C/EBPβ, CCAAT/enhancer-binding protein beta; COPD, Chronic obstructive pulmonary disease; CS, Cigarette smoke; CSE, Cigarette smoke extract; CXCL1, C-X-C motif chemokine ligand 1; CXCL8, C-X-C motif chemokine ligand 8 (also known as IL-8); ELF3, E74-like factor 3; GCs, Glucocorticoids; GR, Glucocorticoid receptor; IL-6, Interleukin-6; IL-17A, Interleukin-17A; IL-17RA/RC, Interleukin-17 receptor A and C complex; IL-1β, Interleukin-1 beta; IL-1R, Interleukin-1 receptor; IκBζ, Inhibitor of nuclear factor kappa-B zeta (encoded by NFKBIZ); mRNA, Messenger RNA; MyD88, Myeloid differentiation primary response 88; NF-κB, Nuclear factor kappa-light-chain-enhancer of activated B cells; Regnase-1, Regulatory RNase 1; SCFAs, Short-chain fatty acids; STAT3, Signal transducer and activator of transcription 3; Th17, T helper 17 cells; TNF-α, Tumor necrosis factor alpha; TNFR, Tumor necrosis factor receptor; TRADD, TNFR1-associated death domain protein; Treg, Regulatory T cells; 3’-UTR, 3’ untranslated region; γδ T cells, Gamma delta T cells.

Independent of classical transcriptional pathways, IL-17A drives corticosteroid resistance in COPD through an mRNA stabilization branch (68). In the resting state, the ribonuclease Regnase-1 mediates the degradation of pro-inflammatory mRNAs including *CXCL1* and *IL6* by recognizing their 3’-UTR stem-loop structures (69). Upon IL-17A receptor binding, ACT1 induces the cytoplasmic translocation of the RNA-binding protein Arid5a, which competitively binds to these 3’-UTR regions, displacing Regnase-1 and blocking its nuclease activity (69, 70). Such de-repression prolongs the half-life of pro-inflammatory transcripts. It acts directly on the cytoplasmic mRNA pool, bypassing the need to initiate new gene transcription. Because this post-transcriptional stabilization operates outside the nucleus, it escapes the transrepression targets through which GR normally suppresses pro-inflammatory gene expression (25, 71) (see **Figure 1**). Because GR transrepression acts at nuclear transcriptional initiation, neither the transcriptional NF-κB/IκBζ loop nor the cytoplasmic ACT1/Arid5a stabilization branch is effectively suppressed by corticosteroids. Compounded by the direct downregulation of the intracellular anti-inflammatory receptors GR and HDAC2 via the IL-17A/NETs axis (16), this dual-layer corticosteroid escape combined with receptor suppression constitutes the pathological mechanism for refractory neutrophilic inflammation in COPD.

### 2.2 IL-17A as an amplifier of inflammation and shaper of the immune microenvironment

Within the COPD airway, the pathogenic contribution of IL-17A extends beyond classical leukocyte chemotaxis to actively modulate the local immune microenvironment. By forming self-amplifying feedback loops and disrupting cellular homeostasis, IL-17A sustains chronic airway inflammation. The following sections detail how this cytokine fosters a steroid-refractory milieu by polarizing neutrophils into specific pathogenic phenotypes, driving systemic Th17/Treg imbalance, and mediating aberrant crosstalk with the respiratory epithelium.

#### 2.2.1 Heterogeneity of neutrophilic inflammation and positive feedback loops

IL-17A initiates neutrophil recruitment and shapes their pathological phenotype to sustain continuous airway inflammation. In classical chemotactic pathways, IL-17A synergizes with environmental stress including PM2.5 or infectious signals, inducing the airway epithelium to release CXCL1 and CXCL8 to establish a concentration gradient that drives neutrophil migration into the airways (18, 33, 72). Epithelial-derived IL-17 family members such as IL-17C exert a synergistic recruitment effect in response to specific pathogens like *Haemophilus influenzae* (73).

Neutrophils in COPD function as critical immunomodulatory nodes. In the microenvironment of porcine pancreatic elastase (PPE)-induced emphysema, IL-17A secreted by lung-resident γδT cells stimulates airway structural cells to release granulocyte colony-stimulating factor (G-CSF) (23, 44). This cytokine drives neutrophil polarization toward a pathogenic Siglec-F^+^ subtype via the JAK2/STAT3 and PI3K-independent mTOR signaling pathways. Compared to classical subsets, Siglec-F^+^ neutrophils secrete more pro-inflammatory cytokines including IL-1β, IL-6, and TNF-α, along with a propensity for NETosis, directly mediating the physical destruction of alveolar structures (23). Whether an equivalent neutrophil subset exists in human COPD lungs is unresolved; addressing this gap will require single-cell profiling of clinical specimens (44). Clinical interventions should avoid pan-neutrophil inhibition strategies that compromise basal host anti-infection immunity. Therapies must target specific hyper-inflammatory subsets or intercept upstream driving signals including the IL-17A/G-CSF axis to resolve refractory airway inflammation while circumventing the risk of secondary infections.

The physical and biochemical interactions between IL-17A and NETs constitute another mechanism for inflammatory cascade amplification. Under CS exposure, activation of the AKT/Nrf2/SLC7A11/GPX4 pathway confers ferroptosis resistance upon neutrophils. By conferring resistance to ferroptosis, this pathway ensures prolonged neutrophil survival, which in turn facilitates the continuous extrusion of DNA-rich NET structures. These web-like structures capture pathogens and extensively sequester IL-17A dimers through physical binding, forming a cytokine reservoir within the local microenvironment (74). Anchored IL-17A retains high biological activity, stimulating adjacent pulmonary fibroblasts to release CXCL1. This chemokine reciprocally recruits additional neutrophils and induces NETosis, establishing a fibroblast-neutrophil-driven IL-17A-CXCL1-NETs positive feedback amplification loop within the lung parenchyma (26).

#### 2.2.2 Dynamic evolution of Th17/Treg immune imbalance

The quantitative and functional imbalance between Th17 and Treg cells characterizes the COPD airway immune microenvironment. Clinical evidence reveals a pro-inflammatory polarization state in the COPD lung: Th17 cells and downstream factors including IL-17A and IL-23 are elevated, while Treg cells are reduced in number and secrete less IL-10 and TGF-β (51, 75). In the asthma-COPD overlap (ACO) subpopulation, an elevated peripheral blood Th17/Treg ratio negatively correlates with the degree of airflow limitation, measured as the forced expiratory volume in one second percentage predicted (FEV_1_% pred). This ratio also positively correlates with fractional exhaled nitric oxide (FeNO) and serum IgE levels, serving as a biomarker for assessing disease activity and potential allergic phenotypes (22). The simultaneous elevation of type 17 and type 2 markers distinguishes ACO from purely neutrophilic or eosinophilic endotypes and reflects concurrent engagement of both immune programs.

IL-17A exacerbates this immune imbalance through three underlying mechanisms. First, competitive antagonism of signaling pathways. The pro-inflammatory network drives sustained STAT3 activation, establishing a competitive counterbalance against the phosphorylation of STAT5, a critical transcription factor for Tregs. Temporal analyses in animal models reveal that defective STAT5 phosphorylation and reduced IL-10 release occur during early disease stages, predisposing Tregs to transdifferentiate into Th17-like cells (51) and facilitating cascade amplification of the STAT3/IL-17 axis (75, 76). Second, epigenetic and transcriptional regulation. Exposure to CS or PM2.5 induces hyperactivation of the Notch signaling pathway, particularly the Notch1/Hes1 axis, exacerbating the Th1/Th17 immunological bias (56). Activation of the aryl hydrocarbon receptor (AhR) exhibits close crosstalk with the Notch axis. The de-repression of Notch signaling resulting from downregulated expression of long non-coding RNAs including the *PVT1* transcript further drives the pathogenic Th17 phenotype (56, 59, 77). Third, mitochondrial metabolic reprogramming. Abnormal store-operated calcium entry (SOCE) within the chronic inflammatory cascade causes intracellular calcium overload and reactive oxygen species (ROS) accumulation, triggering mitochondrial damage. This damage impairs the metabolic adaptability required for CD4^+^ T cells to differentiate into Tregs, forcing a shift toward the Th17 phenotype (78) and contributing to Treg functional exhaustion.

Interventions targeting this immune imbalance exhibit disease-modifying potential. Pharmacological interventions targeting the adenosine A2a receptor (48) or utilizing astragaloside IV to block the CXCR4 axis (47) upregulate Treg levels and inhibit excessive IL-17A secretion. Similar restorative effects are achieved using pharmacologically active natural compounds such as kaempferol (79), or multi-component botanical drugs (e.g., the Lijin formulation), which act as multi-target network modifiers to restore immune homeostasis (46). Modulating the gut-lung axis microbiome (see Section 2.1.2) through SCFA-mediated epigenetic regulation of the *Foxp3*/RORγt balance (37) and systemic exercise rehabilitation interventions (80) demonstrate efficacy in restoring immune balance. Rebuilding local and systemic immune tolerance remains essential for delaying COPD progression.

#### 2.2.3 Core mediators of epithelial-immune cell crosstalk

Airway epithelial cells serve as the primary effector targets of IL-17A and a hub linking immune inflammation to structural remodeling. Beyond inducing the secretion of classical inflammatory mediators such as IL-6 and CXCL8 (72), IL-17A activates IKK to promote the epithelial release of thymic stromal lymphopoietin (TSLP), amplifying local type 2 inflammation and fibrotic signals (81). IL-17A directly downregulates the expression of tight junction proteins including ZO-1 and claudin-1 via the ERK/STAT3 signaling axis. Barrier disintegration is a shared pathological feature across upper and lower respiratory tract inflammation (82, 83) and increases host susceptibility to exogenous pathogens including *Pseudomonas aeruginosa (*42).

IL-17A stimulates pulmonary epithelial cells to generate ROS, triggering oxidative stress and DNA damage (84). Within this high-oxidative-stress microenvironment, IL-17A suppresses the expression of GPX4, the core defense enzyme against ferroptosis, via the ACT1-TRAF6-p38 MAPK pathway. This suppression impairs the cellular capacity to clear lipid peroxides, inducing epithelial cell ferroptosis (85). Cell rupture releases damage-associated molecular patterns (DAMPs) including HMGB1 into the microenvironment. These DAMPs activate neutrophils and promote NET formation, creating a positive feedback loop between cell death and immune hyperactivation (26, 60). IL-17A also operates through the ERK pathway and the NLRP3/Caspase-1 inflammasome to drive gasdermin D (GSDMD)-dependent pyroptosis. These cell death modes mediate the progressive disintegration of the airway epithelium (86–88).

A disintegrin and metalloprotease 9 (ADAM9) acts as an effector molecule bridging inflammation and matrix destruction. Acting synergistically with cigarette smoke extract (CSE), IL-17A upregulates ADAM9 expression in airway epithelial cells. Activated ADAM9 degrades pulmonary elastin and induces alveolar epithelial cell death by cleaving EGFR and VEGFR2, accelerating the progression of emphysema (89). During EMT, IL-17A acts through a paracrine mechanism. IL-17A induces pulmonary stromal fibroblasts to release CXCL12, which drives adjacent epithelial cells via the CXCR4 axis to downregulate E-cadherin and upregulate Vimentin, promoting peribronchiolar fibrosis (28). IL-17A also suppresses basal autophagy in fibroblasts via the PI3K/AKT/mTOR pathway, thereby precipitating the pathological deposition of collagen and altering the mechanical properties of the airway wall.

The pathological evolution of the epithelium and stroma forms a bidirectional feedback loop with the local immune microenvironment. Stimulated epithelial and stromal cells release G-CSF, which sustains neutrophil viability and drives their differentiation toward a pathogenic Siglec-F^+^ phenotype (23). In turn, ROS and NETs released by these effector cells exacerbate epithelial damage and ferroptosis (43). Intercepting the IL-17A-driven ferroptosis-remodeling axis and its immune feedback loop with G-CSF provides an opportunity to reverse airway structural damage in COPD.

### 2.3 Direct association between IL-17A and the core pathological features of COPD

The chronic immune activation driven by IL-17A does not remain confined to the airway mucosa but progressively leads to structural deterioration across multiple lung compartments. Subepithelial fibrosis and collagen deposition increase airway wall stiffness and reduce luminal caliber, elevating airway resistance and contributing to fixed airflow limitation; airway IL-17A expression in COPD patients negatively correlates with FEV_1_/FVC ratio (90). Alveolar septal destruction reduces elastic recoil pressure and diminishes the gas-exchange surface area, reflected by declining diffusing capacity and increased mean linear intercept (Lm); deletion of the *Il17a* gene attenuates these emphysematous lesions in cigarette smoke-exposed mice (91). IL-17A also promotes luminal obstruction through *MUC5AC/MUC5B* hypersecretion combined with impaired mucociliary clearance, generating mucus plugs measurable by CT-based occlusion scores independent of spirometry (15). These processes interact: loss of parenchymal elastic recoil diminishes tethering forces that maintain small airway patency, rendering fibrotic and mucus-occluded airways more susceptible to expiratory collapse. The following sections detail the specific cellular and molecular mechanisms underlying these structural changes.

#### 2.3.1 Airway remodeling: the progression from EMT to irreversible fibrosis

Airway inflammation and structural remodeling are closely intertwined in COPD progression. IL-17A-driven neutrophil recruitment leads to the secretion of neutrophil elastase (NE) and matrix metalloproteinases 9/12 (MMP-9/12). These proteases disrupt ECM metabolic homeostasis (92, 93). These enzymes degrade alveolar supporting structures including the structural prote*in vitro*nectin *(*94) and activate latent TGF-β via proteolytic cleavage, converting local immune activation into a persistent pro-fibrotic signal. IL-17A synergizes with environmental stress including CS to activate pulmonary fibroblasts by upregulating TGF-β. Upon receptor engagement, IL-17A activates the PI3K/AKT/mTOR signaling pathway, suppressing basal autophagy in fibroblasts. This dysregulation impedes the degradation of type I and type III collagen, leading to their pathological deposition within the airway wall. IL-17A-induced mitochondrial dysfunction in fibroblasts further solidifies this pro-fibrotic airway phenotype (95).

Beyond fibrillar collagens, IL-17A promotes the accumulation of small leucine-rich proteoglycans (SLRPs) in the airway wall. In an elastase-induced emphysema model, anti-IL-17 treatment reduced decorin and lumican in airway walls with concurrent TGF-β reduction, although these effects were not replicated in alveolar septa (96). Similar reductions in all three SLRPs were observed in airway walls of a chronic allergic asthma model exacerbated by LPS (97). Although neither model directly recapitulates CS-driven COPD, consistent attenuation by IL-17 blockade points to TGF-β as a shared upstream regulator. Findings in human COPD tissue diverge from these models. Parenchymal versican is reduced, while decorin and biglycan show compartment-specific changes, and overall ECM composition correlates with lung function decline (98). Distal lung fibroblasts from GOLD IV patients also secrete more versican but respond less to TGF-β (99). This contrast likely reflects disease stage, with early IL-17A-driven proteoglycan deposition giving way to proteolytic degradation in advanced disease. Decorin classically acts as a TGF-β antagonist, but excessive accumulation can stabilize collagen fibril organization and increase matrix stiffness in non-pulmonary tissues (100); whether this occurs in the COPD airway has not been tested. Clinically, serum IL-17A levels in biomass smoke-induced COPD (BS-COPD) patients correlate with CT-quantified small airway disease (90). Whether versican is similarly regulated by IL-17A in COPD has not been examined, though its elevation in COPD distal fibroblasts (99) provides a rationale for investigation.

IL-17A-driven ECM degradation also triggers autoimmune amplification through the exposure of cryptic matrix autoantigens. Type V collagen (Col V) is a minor fibrillar collagen normally sequestered within heterotypic collagen I/III fibrils in the lung interstitium (101). NE- and MMP-9/12-mediated fibril disruption exposes Col V neoepitopes, and anti-Col V antibodies have been detected in the peripheral blood of COPD patients and smokers, with elevated levels during exacerbations (101). In patients with end-stage lung disease including COPD, Col V-specific cellular immunity depends on IL-17, IL-1β, and TNF-α rather than IFN-γ, indicating a Th17-dominant effector pathway (102). In a CS-induced murine COPD model, nasal tolerance to Col V attenuated emphysema (reduced Lm), suppressed pulmonary IL-17A, IFN-γ, and IL-6, and promoted Treg differentiation, although elastic fiber degradation was not prevented (101). The IL-17-dependent nature of Col V autoimmunity is further supported by lung transplantation models, where Col V-specific Th17 cells are sufficient to induce rejection pathology and regulatory T cell transfer suppresses this response (103). These data suggest a positive feedback loop in which IL-17A-driven protease release exposes Col V, which activates Th17 responses that further amplify local IL-17A production.

IL-17A-mediated airway remodeling exhibits an independent molecular trajectory in specific environmental exposure phenotypes. This phenotype is prevalent among women in low- and middle-income countries. Signaling crosstalk between IL-17A and the circadian nuclear receptor NR1D1 mediates small airway remodeling in this phenotype. Clinical data demonstrate that serum IL-17A levels are elevated in BS-COPD patients and positively correlate with the severity of small airway disease (90). Biomass smoke exposure prompts IL-17A to downregulate NR1D1 expression in bronchial epithelial cells. The loss of this circadian protein relieves constraints on cell proliferation, accelerating small airway wall thickening and structural solidification. Animal models demonstrate that *Il17a* gene knockout mitigates the progression of small airway remodeling in mice exposed to wood smoke. This mechanism expands the downstream effector network of IL-17A and provides pathological evidence for investigating disease progression in non-smoking populations, particularly women chronically exposed to indoor air pollution.

Beyond extracellular matrix degradation, IL-17A drives EMT through complex paracrine signaling networks. IL-17A stimulates pulmonary fibroblasts to secrete CXCL12, which subsequently targets adjacent bronchial epithelial cells via the CXCR4 receptor (28). Accompanied by ERK1/2 signaling pathway activation, this paracrine process induces the loss of apical-basal polarity in epithelial cells, allowing them to acquire fibroblast-like characteristics. This structural alteration exacerbates airway narrowing. Clinicopathological analyses confirm that airway IL-17A expression levels in COPD patients positively correlate with the severity of small airway disease and the expression of the metalloprotease ADAM9. These levels negatively correlate with airflow limitation parameters including the FEV_1_/FVC ratio (90). Blocking IL-17A signaling alleviates collagen deposition by restoring autophagic flux (29). This blockade curtails the EMT process by downregulating ADAM9 expression (89). The application of JAK1/STAT3 pathway inhibitors including filgotinib downregulates IL-17A levels, reversing fibroblast proliferation and ameliorating pulmonary fibrosis (30). Targeting IL-17A and its downstream network may attenuate the fibrotic and inflammatory components of airway remodeling in COPD (104); however, this approach is contingent upon intervention before irreversible structural obliteration of small airways has been consolidated.

IL-17A also acts on ASM. IL-17A promotes ASM cell proliferation and survival via the ERK1/2 MAPK pathway (105) and drives ASM cell migration through an autocrine growth-related oncogene (GRO)/CXCR2 circuit (106), contributing to increased ASM mass. IL-17A also reprograms ASM into a pro-inflammatory secretory phenotype. It synergizes with TNF-α to stabilize CXCL-8 mRNA via p38 MAPK (107) and with IL-1β to drive CXCL-8 transcription via NF-κB and activator protein 1 (AP-1) (108). Whether IL-17A alone can induce CXCL-8 appears to depend on context (106, 107). At the functional level, IL-17A enhances ASM contractility via RhoA/ROCK2-mediated calcium sensitization, increasing myosin light chain phosphorylation without altering calcium oscillation frequency or amplitude (109). In an experimental ACO model, IL-17 neutralization reduced airway resistance (Raw) while downregulating ROCK1/2 expression (110), linking these cellular mechanisms to measurable physiological improvement. Whether these ASM-specific effects are reproduced in cigarette smoke-based COPD models requires further investigation.

The ASM-specific effects of IL-17A are not invariably coupled to parenchymal pathology. In a chronic ozone-exposure model, IL-17 receptor deficiency selectively abolished airway hyperresponsiveness (AHR) and excessive ASM contractile responses without preventing emphysematous airspace enlargement; Lm increased comparably in IL-17 receptor-deficient and wild-type mice (111). This suggests that IL-17A may affect airway and parenchymal mechanics through partially independent pathways. In a chronic CS model, by contrast, IL-17RA deficiency attenuated emphysema and reduced macrophage-derived MMP-12 expression (112). The contribution of IL-17 signaling to airway versus parenchymal mechanics therefore depends on the nature, intensity, and duration of the injurious stimulus.

These potentially modifiable IL-17A-driven processes must be distinguished from the irreversible structural loss of small airways that characterizes advanced COPD. Micro-CT analyses of explanted lungs have revealed that the total number of terminal bronchioles is reduced by over 70% in very severe COPD, and that this small airway loss precedes the onset of emphysematous destruction in the centrilobular phenotype (13). The surviving airways exhibit wall thickening consistent with active inflammatory remodeling, but also progressive luminal obstruction ranging from mucus-dominated occlusions to complete collapse associated with loss of alveolar attachments; by end-stage disease, virtually all remaining terminal bronchioles are non-aerated (14). This pathological trajectory has direct implications for IL-17A-targeted interventions. Early obstructive lesions driven by active inflammation and mucus hypersecretion may be amenable to anti-inflammatory treatment. Once terminal bronchioles collapse or disappear, however, cytokine-targeted therapy cannot restore the lost architecture. This temporal constraint on disease modification distinguishes COPD from asthma, where airway destruction at this scale does not typically occur and biologic therapies have consequently achieved greater clinical success.

#### 2.3.2 Alveolar destruction and emphysema: specific cell subsets and protease cascades

IL-17A drives the transition from airway inflammation to irreversible alveolar destruction in emphysema. Deleting the *Il17a* gene attenuates emphysematous lesions and ameliorates systemic bone loss, indicating its role in lung-bone axis crosstalk (91). IL-17A secreted by lung-resident γδ T cells (24) stimulates structural cells including the airway epithelium and fibroblasts to release G-CSF (72). In murine emphysema models, this axis recruits bone marrow-derived neutrophils and polarizes them into a long-lived, pathogenic Siglec-F^+^ subset through JAK2/STAT3, PI3K-independent mTOR, and p38 MAPK signaling (23). Whether a comparable activated subset is present in human COPD lungs is not yet established. In other neutrophilic conditions, NETs released by activated neutrophils can bind and retain bioactive IL-17A, forming a local cytokine reservoir (74).

The infiltration of effector cells triggers tissue destruction. IL-17A induces neutrophils to overproduce NE and MMP-9/12, directly degrading alveolar elastic fibers (92, 93). IL-17A accelerates alveolar epithelial cell death by upregulating ADAM9. IL-17A persistently activates the PI3K/AKT/mTOR pathway in target cells to suppress autophagy. This suppression impedes collagen degradation and forces structural cells into premature senescence. Senescent cells amplify the local inflammatory microenvironment by secreting senescence-associated secretory phenotype (SASP) factors (41). IL-17A downregulates the antioxidant enzyme GPX4 via the ACT1-TRAF6-p38 MAPK signaling axis, inducing alveolar epithelial cell ferroptosis (43). DAMPs including HMGB1 released upon cell rupture reciprocally recruit and activate neutrophils. This creates a positive feedback loop driven by lipid peroxide accumulation and protease release, ultimately leading to alveolar septal rupture and emphysema formation (60).

Quantitative analyses using high-resolution computed tomography (HRCT) confirm that IL-17A levels in severe COPD patients positively correlate with small airway disease and wall thickening (5, 90). Blocking the IL-17A/G-CSF axis curtails the recruitment of the pathogenic Siglec-F^+^ subset. Whether IL-17A blockade can preserve alveolar architecture and lung mechanics depends on the experimental paradigm, as outlined below. Microbiome interventions (detailed in Section 2.1.2) based on the gut-lung axis through SCFA-mediated HDAC inhibition or targeting core intracellular nodes including the mTOR pathway and NR1D1 (90), intercept this cascade. These approaches provide disease-modifying strategies for halting emphysema progression.

##### 2.3.2.1 Model-dependent heterogeneity in IL-17A-driven emphysema

The pathogenic role of IL-17A in emphysema differs sharply between experimental models, and these differences directly affect translational interpretation. The porcine pancreatic elastase (PPE) model induces acute protease-driven alveolar destruction over days to weeks, a setting in which IL-17A-mediated neutrophil chemotaxis is a rate-limiting step. Both preventive and therapeutic anti-IL-17 treatment in PPE models reduced Lm and reversed increases in respiratory mechanics including tissue elastance (Htis) and Raw (96). In chronic CS models, however, therapeutic IL-17 neutralization restored Htis and tissue damping (Gtis) but did not alter Raw (113), consistent with observations that CS exposure alone does not significantly increase this parameter in mice (114). This divergence illustrates how the acute proteolytic environment of the PPE model amplifies airway-level effects that are less prominent in chronic smoke-driven disease. Because the PPE paradigm compresses years of human pathology into days, PPE-derived evidence alone may overstate the initiating role of IL-17A in chronic COPD.

Chronic CS exposure models (3–6 months) present a more complex picture. Voss et al. showed that IL-17A deficiency in a 3-month CS model failed to prevent structural destruction and instead caused spontaneous loss of basal lung architecture with reduced elastin expression, indicating that IL-17A is also required for pulmonary structural homeostasis (114). Separately, Negasi et al. demonstrated that CS-induced emphysema in wild-type mice did not persist after smoking cessation unless compounded by a preexisting genetic susceptibility factor (115), illustrating that emphysema progression cannot be ascribed to any single inflammatory mediator.

Nevertheless, genetic and pharmacological evidence from extended CS studies confirms a significant pathological role for IL-17A. Direct *Il17a* deletion in long-term CS models attenuated airspace enlargement, preventing increases in Lm and the destructive index (89, 91). Dual *Il17a/Il17f* deficiency completely prevented CS-induced increases in Lm after 24 weeks of exposure, although the study was limited to histologic analysis (116); the authors attributed the discrepancy with single-knockout studies to compensatory IL-17F signaling. Riani Moreira et al. demonstrated that therapeutic IL-17 neutralization during the final month of an established 6-month CS protocol reversed Lm enlargement and restored Htis and tissue damping (Gtis) (113), providing the first combined morphometric and mechanical evidence of IL-17 blockade efficacy in a chronic CS model. Both parameters reflect the viscoelastic properties of the lung periphery and confirm that IL-17A signaling contributes to the progressive loss of parenchymal compliance in emphysematous disease. The discrepancies across studies likely reflect differences in exposure duration, genetic background, and compensatory IL-17F signaling.

#### 2.3.3 Mucus hypersecretion: transcriptional regulation and the inflammatory microenvironment

Airway mucus hypersecretion characterizes small airway obstruction in COPD. IL-17A induces the transcription of the *MUC5AC* and *MUC5B* genes via the NF-κB and ERK1/2 signaling pathways, promoting goblet cell metaplasia in the airway epithelium (36, 72). IL-17A upregulates the anion exchange protein pendrin (encoded by *SLC26A4*) and suppresses the ciliary transcription factor *FOXJ1*, resulting in airway surface liquid alkalinization and impaired mucociliary clearance (117, 118). Mucin overdrive combined with impaired clearance drives mucus plug formation. Clinically, this mucus hypersecretion exhibits corticosteroid resistance. The physical occlusion of small airways serves as a parameter for assessing disease progression independent of standard lung function metrics (15).

The secretagogue effect of IL-17A relies on synergistic crosstalk with the IL-6/STAT3 signaling axis. In airway epithelial cells, IL-17A induces IL-6 secretion; the secreted IL-6 then activates JAK2/STAT3 signaling in an autocrine/paracrine fashion (119). Activated STAT3 initiates transcriptional programs including the upregulation of SPDEF, the transcriptional regulator of goblet cell differentiation, solidifying this secretory phenotype. In ACO or complex environmental exposure models, IL-17A engages in transcriptional crosstalk with type 2 cytokines including IL-13 via STAT6 and NF-κB. This interaction maintains an inflammation-mucus-remodeling positive feedback loop (120, 121). This crosstalk is clinically most relevant in ACO, where both pathways are co-activated, and underlies the mixed granulocytic inflammation typical of this phenotype. The administration of IL-17A neutralizing antibodies (113) or small-molecule inhibitors including C3G disrupts this synergy (25), reversing exposure-induced epithelial remodeling and luminal obstruction.

The spatial heterogeneity of IL-17A-driven transcriptional reprogramming requires further elucidation. In small airways, the molecular pathways by which IL-17A induces Club cell dedifferentiation and goblet cell metaplasia remain unclear. How this process differs from large airway remodeling patterns requires investigation. Defining these region-specific mechanisms facilitates the identification of treatable traits within early COPD lesions.

**Figure 2:**
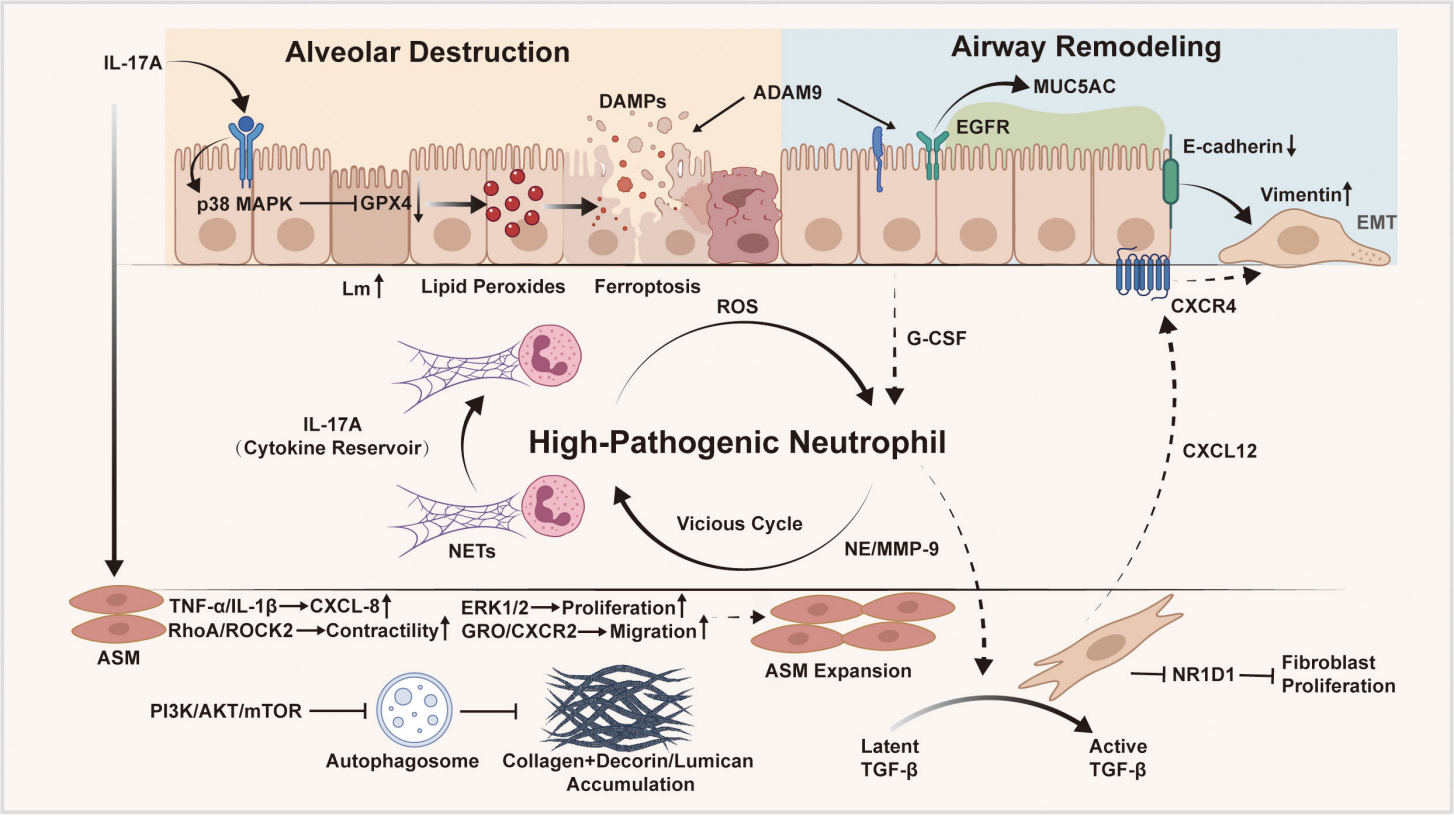
The IL-17A-orchestrated cycle of cellular crosstalk driving pathological airway remodeling and alveolar destruction. A multi-layered interaction network links immune dysregulation to structural deterioration across two distinct anatomical compartments. (Alveolar Destruction Zone) In the ferroptosis zone, IL-17A suppresses GPX4 via the p38 MAPK pathway, culminating in lipid peroxidation and alveolar epithelial cell rupture, leading to increased mean linear intercept (Lm). The released damage-associated molecular patterns (DAMPs) and reactive oxygen species (ROS) further amplify neutrophil activation. ADAM9 also exerts cytotoxic effects in this compartment by cleaving EGFR, contributing to alveolar epithelial death. (Airway Remodeling Zone) ADAM9-mediated EGFR transactivation drives MUC5AC hypersecretion. CXCL12/CXCR4 signaling promotes epithelial-mesenchymal transition (EMT). (Middle Layer) Pathogenic neutrophils form a self-perpetuating cycle by releasing neutrophil extracellular traps (NETs) that physically capture IL-17A. This localized cytokine reservoir sustains neutrophil recruitment and activation. Airway epithelial and stromal cells secrete G-CSF, which polarizes neutrophils toward a long-lived pathogenic phenotype. (Bottom Layer) In the stromal compartment, IL-17A and active TGF-β (cleaved by neutrophil-derived NE and MMP-9) stimulate fibroblasts. IL-17A activates the PI3K/AKT/mTOR axis, which suppresses autophagy; the resulting loss of autophagic clearance permits pathological accumulation of collagen and proteoglycans (decorin, lumican). NR1D1, normally restraining fibroblast proliferation, is suppressed by IL-17A, thereby releasing this brake, leading to airway narrowing. IL-17A also acts on airway smooth muscle (ASM), promoting proliferation (ERK1/2), migration (GRO/CXCR2), pro-inflammatory CXCL-8 secretion (TNF-α/IL-1β synergy), and contractility (RhoA/ROCK2-mediated calcium sensitization), collectively contributing to airway hyperresponsiveness and increased airway resistance. ADAM9, A disintegrin and metalloproteinase domain-containing protein 9; ASM, Airway smooth muscle; CXCL-8, C-X-C motif chemokine ligand 8; CXCL12, C-X-C motif chemokine ligand 12; CXCR2, C-X-C motif chemokine receptor 2; CXCR4, C-X-C motif chemokine receptor 4; DAMPs, Damage-associated molecular patterns; EGFR, Epidermal growth factor receptor; EMT, Epithelial-mesenchymal transition; ERK1/2, Extracellular signal-regulated kinase 1/2; G-CSF, Granulocyte colony-stimulating factor; GPX4, Glutathione peroxidase 4; GRO, Growth-related oncogene; IL-1β, Interleukin-1 beta; IL-17A, Interleukin-17A; Lm, Mean linear intercept; MMP-9, Matrix metalloproteinase-9; MUC5AC, Mucin 5AC, oligomeric mucus/gel-forming; NE, Neutrophil elastase; NETs, Neutrophil extracellular traps; NR1D1, Nuclear receptor subfamily 1 group D member 1 (also known as Rev-erbα); p38 MAPK, p38 mitogen-activated protein kinase; PI3K/AKT/mTOR, Phosphoinositide 3-kinase/Protein kinase B/Mammalian target of rapamycin; RhoA, Ras homolog family member A; ROS, Reactive oxygen species; ROCK2, Rho-associated coiled-coil containing protein kinase 2; TGF-β, Transforming growth factor-beta; TNF-α, Tumor necrosis factor alpha.

Pathological processes ranging from epithelial ferroptosis and NET release to mucus hypersecretion and EMT do not operate in isolation. IL-17A couples these events into an interconnected amplification network, constituting the foundation for progressive airway remodeling in COPD (see **Figure 2**). Evidence from clinical cohorts and experimental models indicates that interrupting this structural and immunological cascade resolves neutrophil-dominated and corticosteroid-resistant inflammation (see **Table 2**).

**Table 2:** Key clinical and preclinical evidence linking IL-17A signaling to neutrophilic inflammation and steroid resistance in COPD.

Evidence type	Pathological feature	Key molecular mechanisms and phenotypes	References
Clinical Evidence	Phenotypic Heterogeneity	Approximately 30% of patients exhibit an IL-17A signature linked to steroid-resistant neutrophilia	(5)
	Disease Severity Correlation	Positively correlates with FEV_1_% decline, small airway remodeling, and elevated FeNO/IgE	(90, 22)
	Th17/Treg Imbalance	Th17 expansion and Treg depletion in blood and lungs inversely correlate with lung function	(51, 18)
Preclinical Evidence	Steroid Resistance	mRNA Stabilization: ACT1/Arid5a displaces Regnase-1, bypassing GR transrepression; HDAC2 Impairment: NETs and oxidative stress suppress HDAC2 and GR translocation; Paradoxical Synergy: IL-17A synergizes with steroids to induce CSF3	(69, 21, 16, 3, 25)
	Specific Neutrophil Subsets and NETs	Drives pathogenic Siglec-F^+^ neutrophil polarization via the γδT-IL-17A/G-CSF axis; Fuels a vicious IL-17A-NETs feedforward loop	(23, 24, 26)
	Airway Remodeling (EMT and Fibrosis)	Drives EMT via the CXCL12/CXCR4 axis and ADAM9 sheddase; Inhibits fibroblast autophagy (PI3K/AKT/mTOR), precipitating collagen accumulation; Suppresses the circadian nuclear receptor NR1D1, promoting abnormal epithelial proliferation	(28, 89, 29, 41, 90)
	Emphysema and Cell Death	Triggers epithelial ferroptosis via ACT1/p38 MAPK/GPX4 axis; amplifies protease (NE, MMP-9/12) release, leading to alveolar destruction	(43, 92, 91)
	Mucus Hypersecretion	Drives MUC5AC/MUC5B hypersecretion via JAK2/STAT3; Upregulates pendrin and downregulates FOXJ1, causing airway surface liquid alkalinization and mucociliary clearance failure	(119, 117, 118)

### 2.4 Therapeutic strategies targeting IL-17A: from mechanistic rationale to clinical translation

As outlined above, COPD pathogenesis involves multiple parallel pathways with extensive functional redundancy. This network architecture distinguishes COPD from diseases in which single-target biologics have succeeded. In psoriasis, IL-17A occupies a less redundant position and anti-IL-17A antibodies achieve high efficacy. In asthma, biologics such as dupilumab benefit from a more hierarchically organized type 2 signaling architecture in which IL-4Rα serves as a functionally convergent target, and validated biomarkers including blood eosinophils and FeNO enable precise patient stratification (45, 122). No equivalent convergent node exists within the non-type 2 inflammatory network in COPD. IL-17A blockade faces redundancy at multiple levels: IL-17F and IL-17A/F heterodimers signal through the same receptor complex (21), IL-17C recruits neutrophils through an independent axis (73), and TNF-α/IL-1β-driven NF-κB activation maintains parallel neutrophil recruitment independently of IL-17A (16, 19). This signaling redundancy, combined with the irreversible small airway destruction that accumulates over decades of disease (13), explains why single-cytokine blockade has failed in unselected COPD populations. The therapeutic value of IL-17A targeting therefore depends on identifying the patient subpopulation in which IL-17A signaling is dominant, and combination strategies addressing compensatory pathways will likely be needed for clinical benefit.

Translating pathophysiological mechanisms into clinical benefits requires addressing the limitations of current COPD treatments. Although targeting the IL-17A pathway provides a rational approach for corticosteroid-resistant neutrophilic inflammation, early clinical trials using non-selective IL-17A blockade failed to improve patient outcomes. To clarify this translational challenge, the following sections examine the specific mechanisms underlying the failure of conventional therapies and initial pan-IL-17A inhibitors, establishing a theoretical basis for emerging biomarker-driven stratification strategies.

#### 2.4.1 Limitations of existing therapies and the scientific basis for targeted intervention

Long-acting bronchodilators combined with ICS form the core regimen for COPD clinical management. While alleviating symptoms, this regimen fails to halt disease progression. This limitation stems from a mismatch between drug mechanisms and COPD pathological phenotypes. Bronchodilators relax airway smooth muscle to improve airflow dynamics without reversing pathological progression. ICS primarily suppresses type 2 inflammation mediated by Th2 cells and eosinophils, with limited efficacy against neutrophilic inflammation in the COPD airway. Long-term ICS application alters the local microbiome, increasing the risk of secondary bacterial infections and pneumonia (2, 123). IL-17A-mediated epithelial cell ferroptosis, EMT, and ECM deposition drive irreversible structural airway alterations inherently refractory to conventional immunosuppressants. The continuous IL-17A-induced secretion of NE and MMP-9/12 causes irreversible physical alveolar damage. IL-17A exacerbates corticosteroid resistance by synergizing with corticosteroids to induce CSF3 expression and disrupting GR homeostasis through the downregulation of GRα and upregulation of GRβ (87). Driven by tissue remodeling and molecular receptor dysfunction, symptom-control strategies fail to reverse airway pathology. Moreover, even targeted anti-cytokine approaches face a temporal constraint: as small airway pathology progresses from mucus-dominated obstruction to complete collapse and physical obliteration (Section 2.3.1), the window for pharmacological intervention narrows. Once alveolar attachments have been destroyed and the airways they support have disappeared, these architectural losses cannot be reversed by anti-inflammatory therapy. This limits the clinical applicability of anti-IL-17A biologics to patients in whom active inflammatory signaling still drives ongoing tissue injury and favors early intervention at a disease stage when obstructive lesions remain potentially modifiable. It also reflects a distinction from asthma, where the airway generally retains its fundamental architecture despite remodeling.

Targeting the IL-17A network offers disease-modifying potential in appropriately stratified subpopulations and at sufficiently early disease stages (93, 104). IL-17A-mediated structural destruction encompasses ADAM9-dependent barrier disintegration and EMT (82, 89). This network maintains chronic inflammation through mechanisms including ectopic lymphoid follicle formation driven by the IL-17/RANKL axis (124). In specific exposure phenotypes including BS-COPD, chronopharmacology broadens the intervention window. Pathological crosstalk between IL-17A and NR1D1 enables interventions in the circadian rhythm network through NR1D1 activation or inhibition of the upstream polarizing signal RORγt (49). Blocking IL-17A and its bypass pathways severs the physical remodeling cascade while restoring epithelial metabolic rhythms and corticosteroid sensitivity. Shifting from simple anti-inflammation to tissue homeostasis restoration may help overcome translational bottlenecks in COPD, provided that intervention occurs before irreversible structural damage has been consolidated.

#### 2.4.2 Direct neutralization strategies: clinical setbacks and the necessity for stratified therapy

Anti-IL-17A monoclonal antibodies are effective in diseases such as psoriasis, but their clinical translation in COPD faces bottlenecks. Phase II clinical trials of pan-IL-17A blockers including CNTO6785 failed to improve FEV_1_ or reduce exacerbation rates in patients with moderate-to-severe COPD (125). Multi-criteria decision analysis (MCDA) reveals the reasons for this setback. Non-selective pan-blockade impairs the basal defense mechanisms of the airway mucosa against intracellular bacteria and fungi, increasing the risk of secondary respiratory infections. The lack of biomarker-based screening dilutes targeted efficacy within a highly heterogeneous population. The Phase III clinical success of the IL-4Rα inhibitor dupilumab in type 2 COPD relied on strict blood eosinophil screening, achieving high MCDA benefit scores (122). Biomarker-driven stratified therapy remains a prerequisite for developing COPD biologics.

This translational dilemma stems from the high endotypic heterogeneity of COPD. Transcriptomic evidence indicates that only approximately 30% of patients exhibit an IL-17A-responsive gene signature (5). This subpopulation shows increased airway microbial colonization and corticosteroid resistance. IL-17A stabilizes pro-inflammatory mRNAs at the post-transcriptional level via the ACT1-Regnase-1 axis. This stabilization bypasses the nuclear transrepression targets of GR (69). This mechanistic escape renders simple corticosteroid dose escalation ineffective for this subpopulation. Interventions must directly block IL-17A or combine with agents capable of restoring HDAC2 activity, including macrolides or low-dose theophylline (3, 6). Sputum cytological analyses indicate that patients with the highest neutrophil proportions exhibit the most refractory corticosteroid resistance (126). In previous unselected trials, the potential benefit to this core subpopulation was masked by data from patients without target-driven disease. Basal IL-17A levels are indispensable for maintaining local pulmonary defense, and this physiological role contributes to the unfavorable risk-benefit profile of non-selective blockade in unselected populations (114).

Beyond heterogeneity, two additional barriers contributed to the CNTO6785 failure. The first is the multi-level signaling redundancy described above. The functional significance of this redundancy is demonstrated by knockout studies: IL-17F expression is upregulated in *Il17a*-deficient mice (127), and dual *Il17a/Il17f* deficiency completely prevented CS-induced airspace enlargement after 24 weeks, whereas single *Il17a* knockout conferred only partial protection (114, 116). Furthermore, NETs physically sequester IL-17A dimers within the local microenvironment, potentially sustaining paracrine signaling even under systemic antibody neutralization (26). The second barrier is the timing of intervention. The trial enrolled patients with established structural disease in whom IL-17A-driven processes may have already consolidated into pathology no longer dependent on continued cytokine signaling. In wild-type mice, CS-induced emphysema stabilizes after smoking cessation unless compounded by genetic susceptibility (115), suggesting that cytokine neutralization alone cannot reverse structurally consolidated damage. However, IL-17 neutralization during ongoing CS exposure partially restores lung mechanics (113), indicating that an intervention window persists while active inflammation continues to drive tissue destruction. These barriers explain why single-cytokine blockade failed in an unselected population without invalidating the target for appropriately stratified patients.

**Figure 3:**
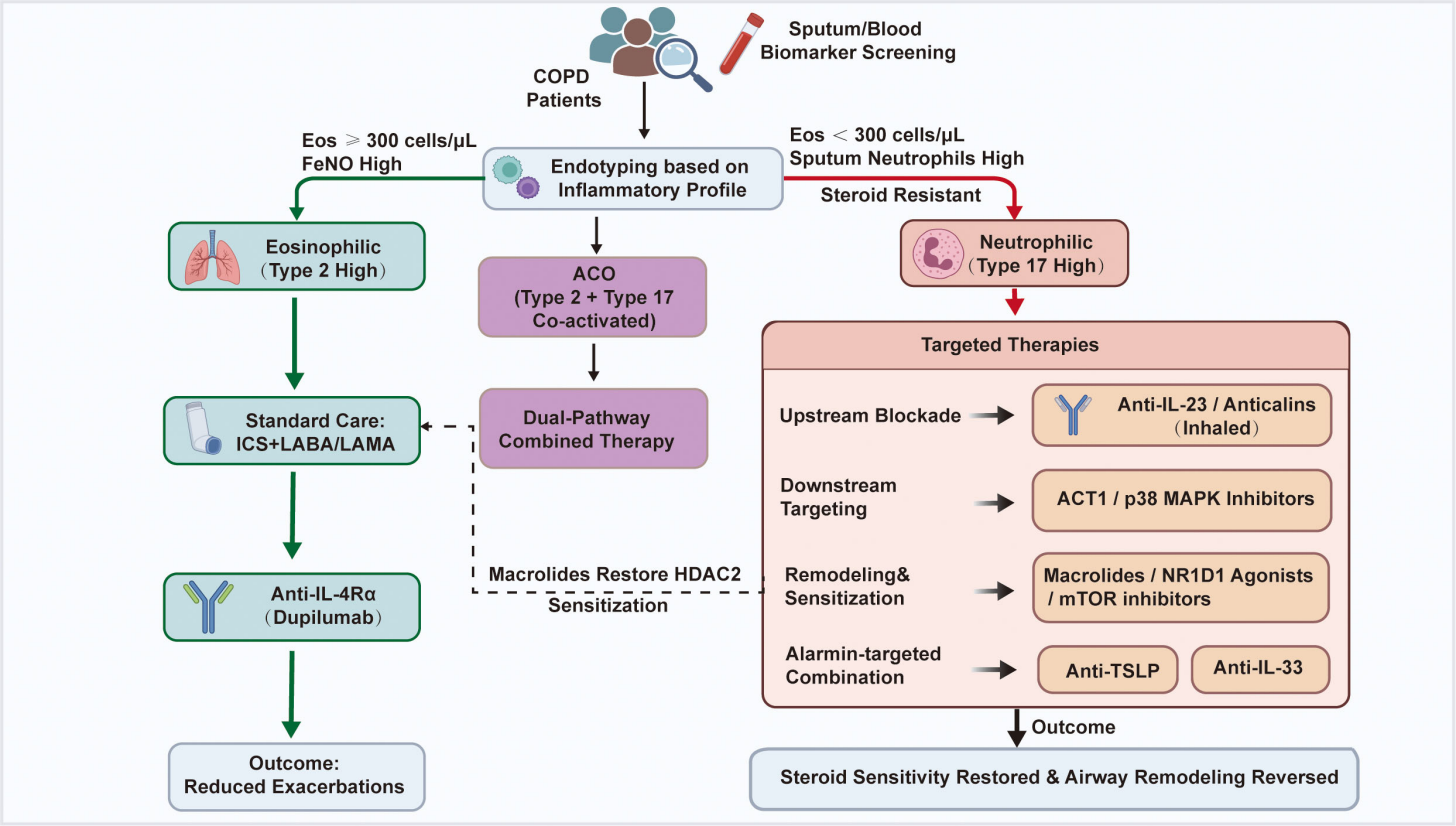
A proposed algorithm for biomarker-driven stratification and targeted therapies in COPD. A clinical decision tree designed to overcome the limitations of non-selective broad-spectrum treatments. Based on biomarker assessments, COPD patients with frequent exacerbations can be stratified into distinct endotypes. (Left Branch) Patients with high type 2 inflammation signatures (blood eosinophils ≥ 300 cells/μL, high FeNO) are optimal candidates for biologics targeting Th2 pathways, including dupilumab (added to ICS+LABA/LAMA in patients with inadequate control). (Right Branch) Patients with non-type 2/IL-17-high signatures (blood eosinophils < 300 cells/μL, high sputum neutrophils) represent a corticosteroid-resistant endotype. For this subpopulation, a multimodal therapeutic strategy is proposed: (1) upstream blockade via anti-IL-23 or Anticalins to safely curtail pathogenic Th17 cells; (2) precision downstream targeting via ACT1 or p38 MAPK inhibitors to uncouple tissue toxicity from host defense; (3) remodeling and combination therapies utilizing macrolides, NR1D1 agonists, and mTOR inhibitors to restore corticosteroid sensitivity, circadian rhythm, and autophagy; and (4) alarmin-targeted combination therapies using anti-TSLP or anti-IL-33 biologics to address upstream epithelial-derived signals that bridge type 2 and non-type 2 inflammation, ultimately achieving disease modification. Of note, patients with ACO may exhibit features of both endotypes, including elevated blood eosinophils coexisting with active IL-17A signaling, and may require dual-pathway strategies rather than allocation to a single branch. The therapeutic window for targeted intervention is limited to the stage when active inflammatory signaling still drives ongoing tissue injury; once irreversible small airway loss has occurred, anti-cytokine therapy alone cannot reverse architectural destruction. Dashed line indicates that macrolides (e.g., azithromycin), within the targeted therapy module of the right branch, can restore HDAC2 activity and re-sensitize neutrophilic patients to inhaled corticosteroids (ICS), thereby allowing reintroduction of the standard ICS+LABA/LAMA regimen as part of an integrated therapeutic strategy. ACO, Asthma-COPD overlap; ACT1, Activating transcription factor 1; Anticalins, Engineered lipocalin-derived binding proteins; COPD, Chronic obstructive pulmonary disease; Eos, Eosinophils; FeNO, Fractional exhaled nitric oxide; HDAC2, Histone deacetylase 2; ICS, Inhaled corticosteroids; IL-17A/IL-23, Interleukin-17A and Interleukin-23; IL-33, Interleukin-33; IL-4Rα, Interleukin-4 receptor alpha subunit; LABA, Long-acting β2-agonist; LAMA, Long-acting muscarinic antagonist; mTOR, Mammalian target of rapamycin; NR1D1, Nuclear receptor subfamily 1 group D member 1 (Rev-erbα); p38 MAPK, p38 mitogen-activated protein kinase; Th2/Th17, T helper 2 and T helper 17 cells; TSLP, Thymic stromal lymphopoietin.

Resolving the current translational bottleneck requires reshaping subject screening strategies. Future clinical trials must abandon pan-population coverage and focus on phenotypically enriched subpopulations (128). Utilizing sputum cytology or transcriptomic tools, trials must identify patients with a high neutrophil burden or high epithelial IL-17A gene signature scores as core inclusion subjects (2). Trial designs must exclude subpopulations sensitive to type 2 targeted therapies, establishing blood eosinophils ≥ 300 cells/μL as a definitive exclusion threshold (45, 122). Precision endotyping based on molecular mechanisms propels anti-IL-17A therapies toward clinical benefit. As proposed in the targeted intervention algorithm (**Figure 3**), delineating the eosinophilic from the neutrophilic endotype utilizing a strict threshold (e.g., blood eosinophils < 300 cells/μL) is the obligatory first step before allocating patients to upstream or downstream IL-17A precision therapies. Current therapeutic development pipelines have shifted from single cytokine blockade toward more diverse and precise strategies (see **Table 3** for a detailed overview of targeted drugs and emerging interventions).

**Table 3:** Current and emerging therapeutic strategies targeting the IL-17A pathway in COPD.

Therapeutic strategy	Representative agents	Key mechanisms and clinical rationale	References
Traditional Pan-Blockade (Clinical Setbacks)	Anti-IL-17A mAbs including CNTO6785	Failed in pan-population trials due to endotypic heterogeneity; highlights the strict imperative for biomarker stratification (e.g., blood eosinophils < 300 cells/μL)	(44, 125)
Upstream Source Containment	IL-23 specific inhibitors including inhalable Anticalins RORγt inhibitors (e.g., PCCR-1)	Deprives pathogenic Th17 cells of survival signals while selectively sparing basal IL-17A, preserving mucosal host defense against infections	(130, 18, 49)
Downstream Precision Inhibition	1. ACT1/p38 MAPK inhibitors (e.g., CHF6297) 2. JAK1/STAT3 pathway inhibitors (e.g., filgotinib)	Precisely severs lethal ferroptosis and EMT signaling cascades while circumventing compensatory feedback failure	(50, 43, 30)
Epigenetic, Metabolic and Circadian Restorers	1. HDAC2 restorers *(Macrolides)* 2. NR1D1 agonists 3. NAD^+^ precursors 4. SCFAs (Gut-lung axis modulators)	Reverses oxidative stress-induced HDAC2 depletion to restore corticosteroid sensitivity; re-establishes circadian homeostasis; and rectifies systemic immunometabolic and epigenetic imbalances	(3, 90, 40, 37, 38)
Pleiotropic Natural Compounds and Botanical Extracts	Baicalein, Resveratrol, Nobiletin, Bufei Yishen formula (BYF), Lijin formula, Shengxian decoction combined with Jinshui Liujun Jian	Multi-component, multi-target profiles; synergistically suppress JAK/STAT, NF-κB, and AhR/Notch networks to restore Treg/Th17 homeostasis	(137, 77, 138, 48, 46, 133)

#### 2.4.3 Upstream blockade: intercepting the immune cascade and epigenetic remodeling at the source

##### 2.4.3.1 IL-23/Th17 axis blockade

Direct neutralization of downstream effector molecules including IL-17A compromises basal host defense, prompting a shift toward upstream blockade of the immune cascade. Targeting the IL-23/Th17 axis inhibits pathogenic IL-17A production. IL-23 maintains the pathogenic Th17 phenotype and synergizes with IL-1β to stimulate IL-17A secretion by effector T cells and ILC3s (52, 129). Inhalable IL-23 inhibitors including the Anticalin protein AcIL-23 validate this upstream interception strategy. Unlike large monoclonal antibodies requiring systemic administration, the low molecular weight and high tissue penetrability of AcIL-23 allow direct airway delivery. In respiratory inflammation models with mixed granulocytic infiltration, inhaled AcIL-23 deprives pathogenic CD4^+^ Th17 cells of survival signals, attenuating neutrophil and macrophage recruitment and reducing AHR. It spares the capacity of local γδT cells to secrete basal IL-17A, maintaining airway mucosal defense against intracellular bacteria and circumventing the risks of pan-IL-17A blockade (18, 130). Clinical observations indicate that continuous application of the anti-IL-23 monoclonal antibody risankizumab in patients with severe COPD during severe acute respiratory syndrome coronavirus 2 (SARS-CoV-2) infection does not worsen respiratory outcomes, supporting the mucosal safety of this target (131).

##### 2.4.3.2 Epigenetic remodeling and natural product-based polypharmacology

Epigenetic remodeling and multi-target interventions offer alternative approaches to reconstruct the immune network. Histone-modifying enzymes regulate chromatin accessibility at specific loci to determine immune cell differentiation. In plasmacytoid dendritic cells (pDCs), the downregulated expression of the histone demethylase gene *JARID1C* increases *Il6* transcription, driving naive T cell polarization toward Th17 cells. Restoring JARID1C enzymatic activity reverses this pro-inflammatory polarization (39). As detailed in Section 2.1.2, gut-derived SCFAs function as epigenetic modulators of the *Foxp3*/RORγt transcriptional balance. Therapeutic strategies aimed at restoring gut microbial diversity and SCFA production therefore represent an upstream approach to attenuating pathogenic Th17 activation in the lungs (37, 38). In addition to specific biologics, naturally occurring small molecules and their derivative botanical extracts exhibit broad-spectrum immunomodulatory properties through polypharmacological mechanisms. For example, the natural polyphenol resveratrol has been shown to reverse the Th17/Treg imbalance by antagonizing the AhR/Notch axis (77). Multi-component botanical preparations, such as the Bufei Yishen extract, mitigate local IL-17A accumulation by suppressing STAT3 phosphorylation while enhancing STAT5 activation, ultimately downregulating RORγt expression (48, 132). Together, these polypharmacological networks (46, 133), alongside targeted small-molecule RORγt inhibitors such as PCCR-1 (49), offer a strategy to block pro-inflammatory cytokine cascades and restore the *Foxp3*/RORγt transcriptional equilibrium. Suppressing pathogenic pathways while restoring basal anti-inflammatory networks provides translational approaches for addressing the immunological heterogeneity of COPD.

#### 2.4.4 Downstream signal blockade and multi-target combination strategies

##### 2.4.4.1 Intracellular signaling node inhibitors

Intervening at specific post-receptor signaling nodes offers an alternative blockade strategy for IL-17A-targeted therapy. The downstream split of ACT1 signaling has functional specificity, where activation of the ACT1-TRAF6 axis mediates the p38 MAPK cascade to drive alveolar epithelial ferroptosis. Utilizing RNA aptamers to generate steric hindrance at the IL-17/IL-17R interface (134) or applying inhaled formulations including CHF6297 to inhibit p38 MAPK (50) attenuates these tissue damage signals while avoiding the broad disruption of immune feedback loops associated with pan-target blockade (65). In airway structural remodeling, targeting the IL-17A-dependent hyperactivation of the PI3K/AKT/mTOR pathway with mTOR inhibitors restores autophagic flux. Inhibiting the JAK1/STAT3 pathway with agents including filgotinib reverses the pro-fibrotic network (30). Targeted interventions against these kinases also correct local metabolic reprogramming, including abnormal glycolysis (135).

##### 2.4.4.2 Metabolic and epigenetic restoration

Correcting metabolic disorders offers another route for controlling inflammation. A randomized controlled trial (RCT) demonstrates that daily supplementation with 2 g of the NAD^+^ precursor nicotinamide riboside (NR) for 6 weeks in stable COPD patients elevates whole-blood NAD^+^ concentrations while inducing a 52.6% reduction in sputum IL-8, an effect persisting for 12 weeks post-cessation (40, 136). Restoring intracellular NAD^+^ reservoirs via metabolic reprogramming attenuates aging-associated epigenetic disorders and immune cell hyperactivation, offering a non-immunosuppressive strategy for intervening in the IL-17A/IL-8-driven neutrophilic inflammatory network.

##### 2.4.4.3 Combined immunosuppressive and anti-microbial therapies

Because of the signaling redundancy in the COPD inflammatory network, multi-target combination therapies offer high clinical translational value. To address corticosteroid resistance in neutrophil-predominant patients, macrolide antibiotics including azithromycin exhibit sensitizing synergy. By inhibiting the PI3K/AKT signaling pathway, these agents restore the expression and activity of HDAC2, reversing functional deficits induced by oxidative stress (3, 6, 123). The combined application of phosphodiesterase 4 (PDE4) inhibitors such as roflumilast with corticosteroids synergistically suppresses IL-17A release and restores HDAC2 activity (137). In animal models of acute exacerbation of COPD (AECOPD) complicated by *Pseudomonas aeruginosa* infection, combining anti-IL-17A therapy with antibiotics such as ciprofloxacin demonstrates lung function protection superior to antibiotic monotherapy. This combination circumvents the secondary infection risks associated with standalone immunosuppressants (42).

##### 2.4.4.4 Natural compounds and traditional botanical formulations

Natural products and their compound formulas provide complements for systemic immune intervention through their network-regulating characteristics (46). When delivered via nanocarriers, the flavonoid baicalein dually inhibits the JAK/STAT and NF-κB pathways (138). Nobiletin inhibits multiple inflammatory mediators (139). Ginsenoside Rg1 attenuates airway fibrosis via the TGF-β/Smad pathway, while tanshinone derivatives and astragaloside IV block the MAPK/HIF-1α and CXCR4 axes to delay airway fibrosis and remodeling (47, 92). Combining these multi-target network modifiers with circadian regulators including NR1D1 agonists overcomes the limitations of single-target drugs, establishing a foundation for formulating combined regimens that integrate specific signal blockade with systemic homeostasis restoration.

##### 2.4.4.5 Combination with upstream alarmin-targeted biologics

Combining IL-17A pathway inhibitors with upstream alarmin-targeted biologics offers another therapeutic avenue. IL-17A induces epithelial TSLP release through IKK activation (81), while IL-33 from damaged epithelium activates type 2 responses through ILC2s and non-type 2 responses through ST2-expressing macrophages.

Several Phase 2a trials have now tested alarmin inhibition in COPD. The anti-TSLP antibody tezepelumab reduced exacerbations in patients with baseline eosinophils ≥150 cells/μL but did not lower neutrophil counts (140), indicating that anti-TSLP alone cannot resolve neutrophilic inflammation. Itepekimab (anti-IL-33) reduced exacerbations only in former smokers (141). Tozorakimab, which blocks both reduced and oxidized IL-33, was effective regardless of smoking status and also reduced mucus plugging on CT (142). None of these agents resolved steroid-resistant neutrophilic inflammation, which is the rationale for pairing them with IL-17A-targeted therapy.

The clearest clinical context for such combinations is ACO, in which type 2 and type 17 axes are co-activated (22). In a murine ACO model, anti-IL-17 treatment reduced airway hyperresponsiveness, ECM remodeling, and oxidative stress, and also lowered tissue eosinophils and IL-5 expression (110). This pattern suggests that IL-17A amplifies type 2 cell recruitment, possibly through epithelial TSLP release (81). Because the elastase component of this model produces acute proteolytic injury rather than chronic inflammatory remodeling, the findings need confirmation in CS-driven ACO models before any clinical extrapolation. The coexistence of elevated eosinophils and active IL-17A signaling in ACO also challenges the binary stratification shown in **Figure 3**, and composite biomarker panels combining sputum cytology with IL-17A gene signatures will be needed to assign endotypes accurately. The infection risk of simultaneously suppressing type 2 and type 17 mucosal immunity will need to be evaluated before clinical translation.

## 3 Conclusions and future perspectives

The pathological evolution of COPD is driven by an integrated feedback loop between immune-inflammatory networks and airway structural remodeling. Within this axis, IL-17A is one of several mediators rather than a sole driver, and effective targeting depends on biomarker-driven stratification rather than indiscriminate blockade. Such intervention is most likely to succeed when applied early, before terminal bronchiole loss imposes architectural limits that anti-cytokine therapy cannot reverse. Mechanistically, IL-17A drives persistent airway inflammation by recruiting metabolically reprogrammed neutrophil subsets such as Siglec-F^+^ cells; promotes EMT via the CXCL12/CXCR4 axis, induces epithelial ferroptosis, and ultimately contributes to airway fibrosis and mucus plugging. This inflammation–metabolism–remodeling axis underlies both the corticosteroid resistance and the progressive lung function decline observed in IL-17A-driven endotypes.

Future progress depends on aligning mechanistic insight with translational tools. Single-cell multi-omics and spatial transcriptomics will define spatiotemporal immunological heterogeneity at the lesion level, while lung-on-a-chip platforms reproducing the cigarette smoke microenvironment (143) enable high-throughput screening of upstream interceptors (Anticalins, RORγt inhibitors), downstream node-specific inhibitors (ACT1, p38 MAPK), and adjunctive modulators (NR1D1 agonists, NAD^+^ precursors, SCFA-based microbiome therapies). Integrating these technologies with biomarker-enriched clinical trials will be essential to move IL-17A-targeted therapy from mechanistic concept to disease modification in COPD.

### 3.1 Clinical implications

The findings reviewed here have direct implications for the clinical management of COPD. IL-17A-driven inflammation is most relevant in patients with steroid-resistant, neutrophil-predominant disease, who represent approximately 30% of the COPD population based on transcriptomic data. The failure of non-selective IL-17A blockade (CNTO6785) in unselected populations confirms that biomarker-based enrichment is a prerequisite for future trials. Persistently high sputum neutrophils combined with blood eosinophils below 300 cells/μL identify the most likely candidates, while patients with eosinophils ≥ 300 cells/μL and elevated FeNO should instead be considered for type 2-targeted biologics such as dupilumab.

For neutrophilic, IL-17A-driven patients, escalating ICS doses is unlikely to confer benefit, because IL-17A drives corticosteroid resistance through post-transcriptional mRNA stabilization that bypasses GR transrepression. Adding macrolides or low-dose theophylline to restore HDAC2 activity is a more rational approach within current treatment options. Patients with ACO, in whom type 2 and type 17 pathways are co-activated, may require dual-pathway strategies, although dedicated clinical trials are currently lacking. Until validated companion diagnostics (sputum cytology panels or epithelial gene-signature scores) are available, treatment decisions for the IL-17A-high endotype will continue to rely on composite clinical and laboratory features rather than a single confirmatory biomarker.
